# Continuous Laparoscopic Closure of the Linea Alba with Barbed Sutures Combined with Laparoscopic Mesh Implantation (IPOM Plus Repair) As a New Technique for Treatment of Abdominal Hernias

**DOI:** 10.3389/fsurg.2017.00062

**Published:** 2017-11-02

**Authors:** Reiko Wiessner, Thomas Vorwerk, Claudia Tolla-Jensen, Alexander Gehring

**Affiliations:** ^1^Department of General and Visceral Surgery, Boddenkliniken Ribnitz-Damgarten, Ribnitz-Damgarten, Germany

**Keywords:** abdominal Hernia, barbed suture, laparoscopic component separation, IPOMplus

## Abstract

Despite extensive experience and significant reduction of complications in recent years, laparoscopic treatment of complex abdominal hernias is a challenge even for the experienced endoscopic surgeon. Patients with severe incisional hernias or symptomatic rectus diastasis benefit from the closure of the linea alba as a morphological and physiological reconstruction of the abdominal wall followed by mesh implantation. Occasionally, an additional component separation is necessary. In open surgery, this is associated with very large wound areas, postoperative seromas, poor wound healing and, in the worst case, mesh infections. To avoid these complications, we operate these complex reconstructions completely endoscopically. Our concept is based on a laparoscopic closure of the linea alba through an ongoing, barbed non-resorbable 1–0 suture (polybutester) and final reinforcement by an intraperitoneal-onlay mesh (IPOM-Plus). For the treatment of complex abdominal hernias with a width of more than 10 cm, we performed an endoscopic anterior bilateral component separation. This allows the surgeon to combine the advantages of the open abdominal wall reconstruction with those of laparoscopic hernia repair. Between May 2015 and June 2017, we treated 42 patients with abdominal hernias by laparoscopic continuous hernia defect closure and complementary mesh implantation, whereby a complex reconstruction with additional endoscopic anterior component separation was performed in five patients. In this article, we will present this innovative technique of endoscopic/laparoscopic hernia repair in complex abdominal hernias.

## Introduction

The treatment of severe abdominal wall hernias is a challenge, both conventionally and laparoscopically. The size of the hernia gap, in particular with the classical IPOM as bridging method, has a great influence on the shear forces to the mesh and, therefore, on possible bulging phenomenon and the forces acting on the fixation points (1). In an abdominal wall model, it could be proved that the mesh overlap should be proportional to the size of the hernia defect (2). The larger the break gap, the more the overlapping of the inserted mesh should be (3). Therefore, the standard IPOM reaches its limits with large defects. As a consequence, in addition to functional morphological aspects of the abdominal wall reconstruction, the closure of the linea alba has the advantage that a sufficient overlap with a mesh is again possible. In addition to the well-known advantages of laparoscopic care (lower wound infection and seroma rate, shorter hospital stay) compared to retromuscular hernia repair (Rives–Stoppa-sublay-technique), this method, which was one of the first described by Chelala, does not lead to the destruction of intact muscle compartments or of the segmental nerve innervation (1, 4, 5). In cases with very large hernia defects, however, the technique reaches its limits. We have established a surgical technique in our clinic where patients receive a laparoscopic closure of the linea alba with an intracorporal, continuous, non-resorbable, self-sustaining suture, and final reinforcement by an intraperitoneal-onlay mesh. For treatment, large abdominal wall defects first receive a bilateral endoscopic anterior component (EAK) separation followed by the IPOM Plus technique.

## Methods

All patients agreed to the operative procedure and to the registration of their data in the German Herniamed Register. The preoperative diagnostics of the patients with a large abdominal wall defect included a CT scan of the abdomen. The hernia gap or rectus diastasis, the linea semilunaris, as well as the primary insertion site in the area of the external oblique muscle below the ribcage, were accurately measured using the CT images or using ultrasound and, if necessary, marked on both sides in order to determine the optimum access for the endoscopic component separation. Special preoperative preparation was not necessary. For the complex hernias, the anesthesiological preparation usually includes the placement of a periduralcatheter for postoperative analgesia. All patients received a perioperative antibiotic prophylaxis with a second-generation cephalosporin and were placed in a supine position with arms outstretched on both sides. The surgeon and first assistant stood for endoscopic component separation on the respective operating side; for the laparoscopic IPOM-Plus technique on the left side of the patient contralateral to the hernia gap. The surgical nurse stood on the opposite side. The monitor was near the feet at the beginning when we started with EAK, and for the laparoscopic part of the operation then on the right side of the patient.

**Figure 1:**
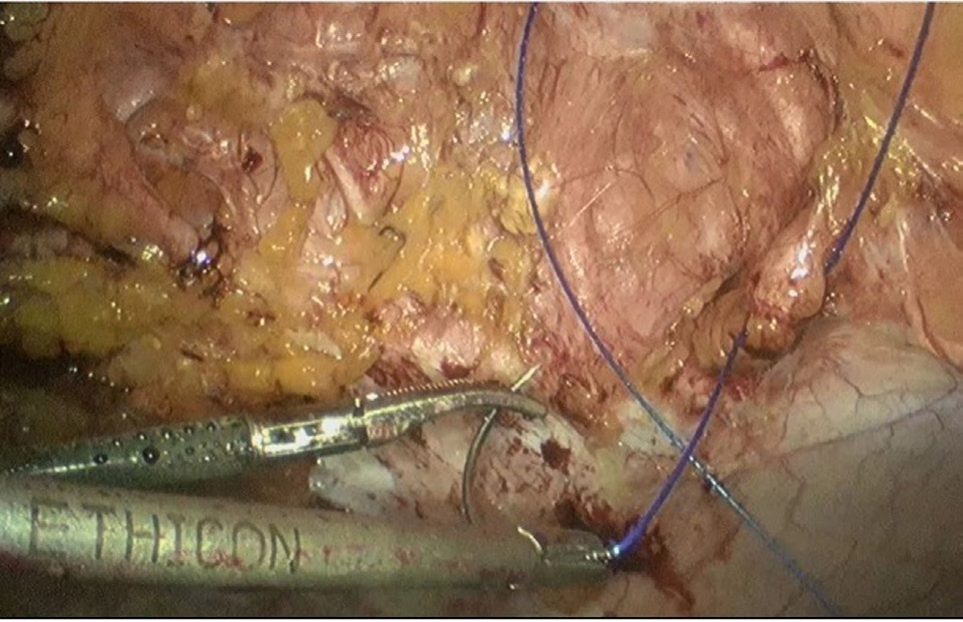
Closure of the midline or linea alba through a continuous, self-sustaining, non-resorbable V-Lok 1./0 suture.

**Figure 2:**
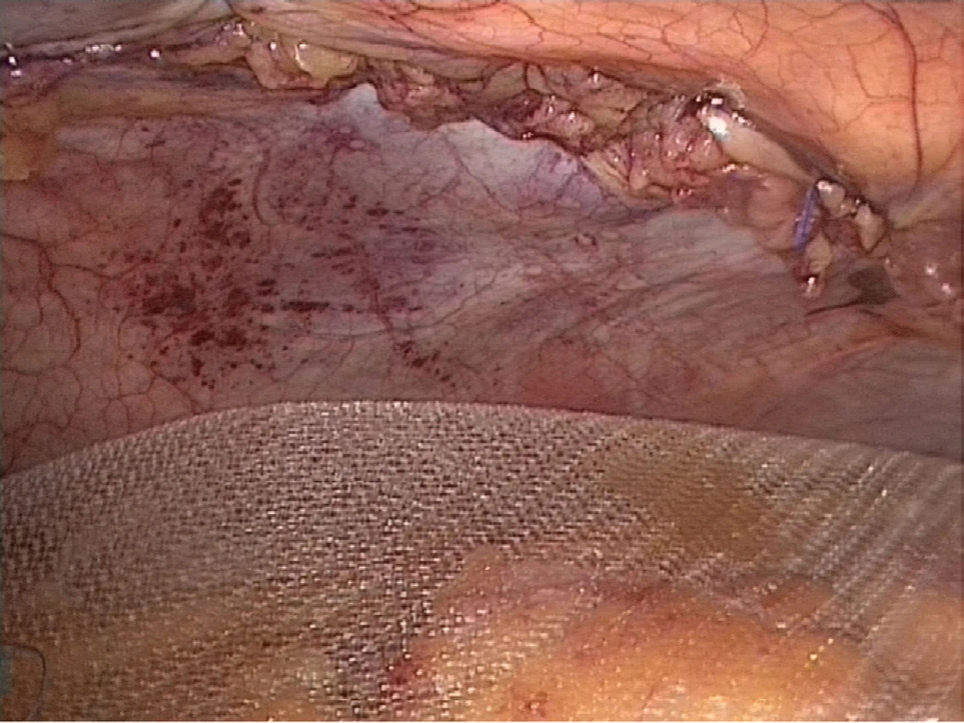
Complete closure of the linea alba prior IPOM mesh implantation.

To perform the IPOM Plus technique, a capnoperitoneum (12–15 mmHg) was built up. The primary trocar was set in the left upper abdomen (*via* minilaparotomy), two other trocars were set in the left middle and lower abdomen. If necessary, adhesiolysis was first performed. The hernia gap or rectus diastasis was deperitonealized in order to prevent a seroma formation after closure and to facilitate the healing of the hernia defect after the laparoscopic suture. This also included the transection of the ligamentum teres hepatis. The laparoscopic closure of the hernia defect or rectus diastasis was then performed with a continuous, self-sustaining, non-resorbable V-Lok 1./0 suture (Medtronic GmbH, Meerbusch, Germany) with a random distance of 1.5 cm from stich to stich and a random distance from 1.5 cm to the middle line. Depending on the length of the hernia gap, at least two, in some cases three sutures were required (Figures 1 and 2). In addition, we reduced the intraabdominal pressure to approx. 8 mmHg and adapted the margins of the hernia gap by applying moderate external pressure. As a last step, the laparoscopic IPOM was performed using the usual technique. Therefore, 2 or, respectively, 4 tranfascial sutures were used for aligning the mesh with non resorbable sutures (*Ti-Cron*™ 0, Medtronic). The final fixation of the mesh took place with resorbable tacks (30–90 *AbsorbaTacks*™, Medtronic) in double crown technology. Due to the closure of the linea alba, an overlap of the mesh of more than 7 (10) cm was easy to reach on both sides (Video S1 in Supplementary Material).

**Figure 3:**
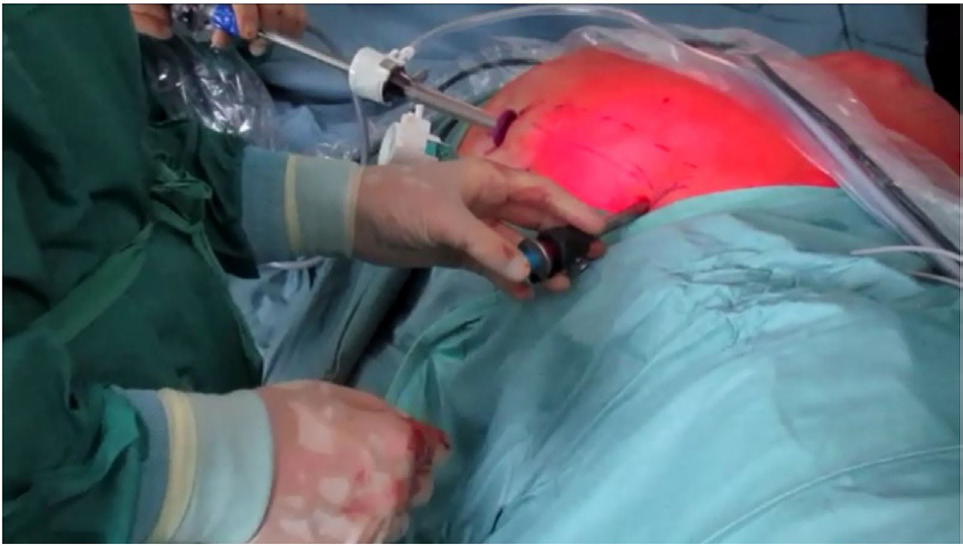
Trocar position for the endoscopic anterior component separation on the right side.

**Figure 4:**
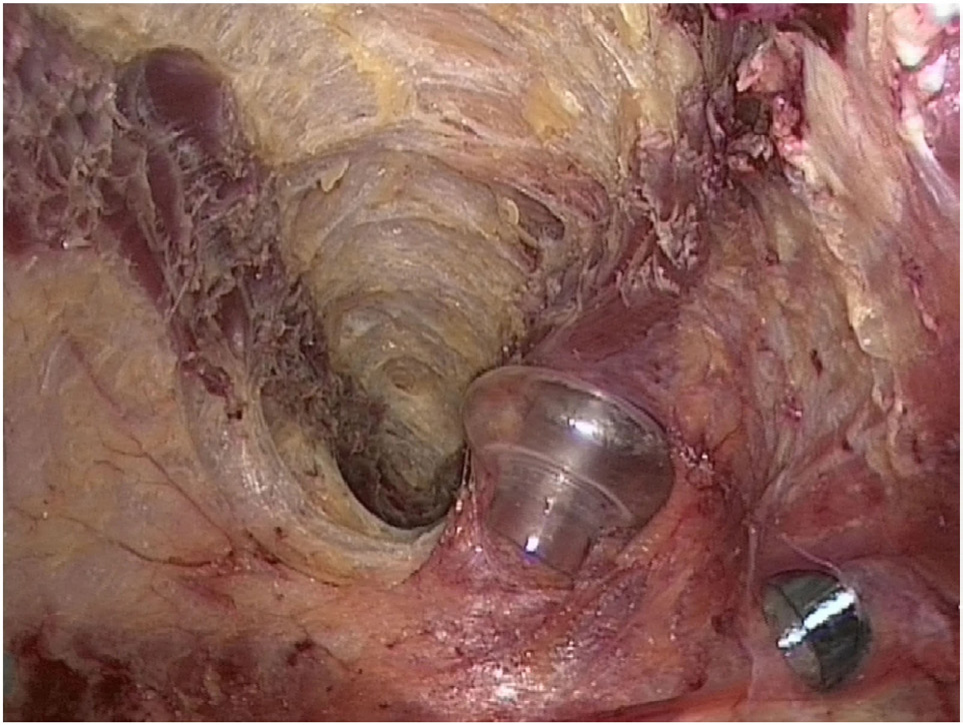
Approximately 5 cm release of the oblique external muscle at the level of the costal arch.

In five patients with complex abdominal hernias, we combined the IPOM Plus technique with the endoscopic anterior component separation in the first step of the operation, and it was carried out as described by Rosen et al. (6). For this purpose, three trocars—a 12 mm balloon-trocar, a reusable 10 mm trocar, as well as an 11 mm one-way trocar were used (Figure 3). For the endoscopic component separation, we used a pressure of 10–12 mmHg CO_2_. In doing so, we abandoned the balloon-dissector used by Rosen and created the space between the internal oblique muscle and the external oblique muscle by digital preparation and then with the optics. Due to the opening of the external aponeurosis, a release of 5–6 cm could be achieved for each side, significantly more than for the posterior component separation (Figure 4). A tissue-sealing device should be used for the separation of the muscular portion of the external aponeurosis in the region of the ribs. In the majority of cases, there is no need to use redon-drainages (Video S2 in Supplementary Material).

Our technique was presented at the 134th Congress of the German Society of Surgery March 21st 2017 in Munich.

## Results

In summary, we have operated on 42 patients (25 men and 17 women, mean age = 64 years, range was 41–82 years) with a direct laparoscopic closure of the linea alba and subsequent IPOM-technique. The patient characteristics and operative data of all patients are presented in Table 1. The mean body mass index was 31.8 kg/m^2^ (range 22.7–48.4 kg/m^2^) and the mean fascia defect had a size of 39.5 cm^2^ (range 3–253 cm^2^). In five patients with a large abdominal wall defect (mean fascia defect 140 cm^2^, range = 56–253 cm^2^), with a European Hernia Society classification of W3 (wall defect >10 cm), the above-described endoscopic anterior component separation was performed additionally (Table 2). There was one intraoperative complication. One patient had an enterotomy in the small intestine during adhesiolysis. This was closed by a seromuscular suture. The postoperative healing process was inconspicuous in all five patients. There was no secondary bleeding, no formation of seromas or wound infections in all 42 patients. During a mean follow-up period of 10 (range = 1–24) months, none of the patients showed recurrence or a bulging phenomenon. Thus far, a follow-up after 12 months has been performed in 42.9% (*n* = 18) of the patients. In one patient, one transfascial suture had to be removed after 4 months due to chronic pain symptoms. One patient developed a trocar hernia after 9 months. All of our patients were very satisfied with the result.

**Table 1:** Patient characteristics and operative data.

Total number of patients	42
Male	25
Female	17
Age (years)	
Mean	63.5
Range	41–82
BMI (kg/m^2^)	
Mean	31.8
Range	22.7–48.4
Incisional hernia	24 (57.1%)
Patients with prior abdominal surgeries	28 (66.7%)
Patients with prior hernia repair	8 (19%)
ASA classification (%)	
ASA I	0
ASA II	33.3
ASA III	61.9
ASA IV	4.8
Comorbidities (e.g., DM)	
Mean	3
Range	1–8
Size of fascial defect (cm^2^)	
Mean	39.5
Range	3–253
SD	53.9
Size of synthetic mesh (cm^2^)	
Mean	327.2
Range	81–600
SD	162
Operating time (min)	
Mean	92.4
Range	47–255
SD	45.1
Intraoperative complications	1
Length of hospitalization (days)	
Mean	4.6
Range	1–27

*BMI, body mass index; DM, diabetes mellitus; ASA, American Society of Anesthesiologists*.

**Table 2:** Patient with endoscopic anterior component separation, direct laparoscopic closure of the linea alba, and following IPOM-technique.

Total number of patients	5 (male)
Age (years)	
Mean	60.4
Range	52–73
BMI (kg/m^2^)	
Mean	33.3
Range	26–42
Patients with prior abdominal surgeries	5
Patients with prior hernia repair	2
Comorbidities	
COPD	1 (20%)
Art. hypertension	3 (60%)
DM	3 (60%)
Tobacco use	3 (60%)
Cardiomyopathy	1 (20%)
Chron. pancreatitis	1 (20%)
OSA	2 (40%)
Size of fascial defect (cm^2^)	
Mean	140
Range	56–253
Size of synthetic mesh (cm^2^)	600
Synthetic mesh material	
Polyester	2 (40%)
PVDF	3 (60%)
Operating time (min)	
Mean	183
Range	138–255
Intraoperative complications	1 (20%)^a^
Length of hospitalization (days)	
Mean	7
Range	4–10

*BMI, body mass index; DM, diabetes mellitus; COPD, chronic obstructive pulmonary disease; OSA, obstructive sleep apnea syndrome; PVDF, polyvinylidene fluoride*.

*^a^Enterotomy during adhesiolysis*.

## Discussion

With the use of this method in large abdominal wall hernias or symptomatic rectus diastasis, the advantages of the open functional-morphological abdominal wall reconstruction, as recommended especially for younger patients (4), can be combined with the advantages of the laparoscopic hernia supply in IPOM-Plus technique. This has advantages in terms of recurrence and complication rate, such as seroma formation and the bulging phenomenon, compared to the conventional IPOM technique without midline occlusion (7). In particular, the meta-analysis of Tandon et al. has demonstrated significant advantages for the fascial closure before laparoscopic hernia repair for adverse hernia site outcomes (recurrence, mesh eventration, tissue eventration, bulging) and postoperative seroma formation (8).

The use of a non-absorbable self-sustaining suture makes the implementation of the laparoscopic midline closure technically easier than the previously performed seam methods (9, 10). In the literature to date, there have been two articles that combine the endoscopic component separation technique with the IPOM-Plus technique (11, 12). Orenstein et al. performed the endoscopic anterior component separation technique in two patients, but subsequently closed the hernia defect in extracorporeal suture technique (11).

Due to the bilateral endoscopic anterior component separation, a release of more than 6 cm is achievable, especially at the umbilical level, so that even large defects can be closed by an intracorporal laparoscopic suture without tension. In this context, we see the advantage in comparison to Streh’s laparoscopic triple-step method (12). He performs the closure of the midline as a dorsal component separation in the Milburn technique (13) or by using a continuous suture of the central line by externally guided resorbable PDS sling completed by an IPOM mesh reinforcement. The lateral release is significantly smaller after dorsal component separation compared to (endoscopically) anteriorly performed separation, so that larger defects can’t be closed in a stress-free manner. In addition, the correct approach at the border between the rectus abdominis muscle to the fascia of the transversus abdominis muscle, to maintain the perforator vessels and the nerves, cannot always be presented laparoscopically. Furthermore, we see more advantages in the use of a non-resorbable suture compared to a long-term-resorbable suture, which is inserted laparoscopically without additional incision of the skin. For example, Lambrecht et al. have demonstrated in a prospective randomized controlled study that the use of a resorbable suture does not lead to any improvement in long-term results and complications (14).

Recent works are concerned with the differences between the conventional transversus abdominis release (TAR) and the robotic TAR (15). The average operative time was significantly longer in the robotic group, but the blood loss and the lower systemic complications as well as the length of hospital stay were significantly lower. The authors around Novitsky et al. recommend the selective application of the robotic TAR.

In addition to the type of the middle line occlusion, the intraperitoneal mesh implantation procedure itself is currently the subject of controversial discussions. Due to rare but possible complications, such as adhesions, mesh fistulation, and mesh migration into the bowel, many surgeons reject the intraperitoneal mesh implantation and instead favor the open retromuscular mesh placement (16). On the other hand, Mercoli et al. report over 417 patients who were monitored over a 10-year period and who had a low recurrence rate of 9.8%. The authors conclude that the standardized laparoscopic IPOM technique is the reference procedure for the treatment of ventral and incisional hernias (17).

This has also been confirmed by registration data from the ACS NSQIP database. Ecker et al. examined 13,567 patients after elective ventral hernia repair (18). A total of 9,228 patients (69%) were operated on in open procedures and 4,339 patients (31%) laparoscopically. The multivariate analysis showed significant advantages of laparoscopy with regard to the occurrence of wound complications, reoperations, blood transfusions, and formation of an ARDS. In addition, fewer reoperations were associated with a hernia recurrence and thus with significantly lower costs (18). This work demonstrates once again how important and necessary databases are in addition to prospective-randomized studies for the assessment of operative procedures. Despite all the discussions concerning the intraperitoneal mesh placement, as well as the different materials used, the use of laparoscopic mesh implantation (IPOM) for primary and incisional hernias is established, safe and in many respects superior to open techniques.

Another innovative pathway for the supply of umbilical/epigastric hernia with rectus diastasis as well as incisional hernias was undertaken by Köckerling et al. and Reinpold with the ELAR plus (endoscopic assisted linear reconstruction plus mesh augmentation) and MILOS (Mini Less Open Sublay Technique) technique (19, 20). Both methods reconstruct the abdominal wall from the outside *via* a small access with similar advantages as the laparoscopic technique: fewer wound infections and pain, shorter hospital stays, good cosmetic results, and rapid postoperative resilience. In the case of the ELAR plus procedure, the implantation of the mesh is performed in an onlay position (epifascial) as augmentation after reconstruction of the linea alba, which is generally associated with a higher recurrence rate, so that we see advantages for our technique. Long-term results for the ELAR plus procedure have not yet been published. The 30-day follow-up shows very good results with a very low complication rate for wound healing, pain, and painkiller consumption (19).

A further development of the MILOS technique is the endoscopic mini/less open sublay technique (EMILOS). Schwarz et al. demonstrated the retromuscular implantation of a 20 cm × 30 cm mesh without opening the abdominal cavity *via* an access of 5.2 cm in 25 patients with midline umbilical, epigastric, or incisional hernias with coexisting rectus diastasis (21). The authors present excellent results and follow the trend of not placing the mesh in the abdomen.

A different method of the laparoscopic but extraperitoneal mesh placement has been described by Yang and Tung (22). With their preperitoneal onlay mesh repair (PPOM) for ventral abdominal wall and incisional hernia technique, they combine the laparoscopic technique with a preperitoneal mesh insert. Schroeder et al. developed the laparoscopic transperitoneal sublay mesh repair for small and medium-sized hernias and compared 43 patients with their new technique with 50 patients who underwent a conventional open operation using the Rives and Stoppa technique (23). There were no significant differences between the two groups. The laparoscopic sublay repair could be simplified by combining it with a self-gripping mesh (24). Moore et al. performed the laparoscopic extraperitoneal stapled sublay mesh technique on 10 patients. By means of additional laparoscopic posterior component separation in three patients, large defects could also be closed. Compared to the conventional IPOM technique, these patients experienced both less postoperative pain and less analgesic consumption (24).

Our previous follow-up studies show similarly good results. Here, a regular follow-up will show whether a low recurrence rate goes with good quality of life in long term. We do not see our technique in competition with the well-known innovative surgical procedures for the reconstruction of the abdominal wall, but rather as an additional alternative for the appropriate patient in the sense of a “tailored approach.”

## Author Contributions

RW has designed the study. RW has written the manuscript. CT-J and AG have revised the manuscript. RW and TV have equally contributed to data collections and data analysis.

## Conflict of Interest Statement

The authors declare that the research was conducted in the absence of any commercial or financial relationships that could be construed as a potential conflict of interest.

## Supplementary Material

Video S1 Continuous laparoscopic closure with barbed sutures combined with mesh implantation (IPOM Plus repair).

Video S2 Endoscopic anterior bilateral component separation.
